# Getting on the same page: Communication, patient involvement and shared understanding of “decisions” in oncology

**DOI:** 10.1111/hex.12592

**Published:** 2017-06-21

**Authors:** Aaron L. Leppin, Marleen Kunneman, Julie Hathaway, Cara Fernandez, Victor M. Montori, Jon C. Tilburt

**Affiliations:** ^1^ Knowledge and Evaluation Research Unit Mayo Clinic Rochester MN USA; ^2^ Division of Health Care and Policy Research Mayo Clinic Rochester MN USA; ^3^ Department of Medical Psychology Academic Medical Center University of Amsterdam Amsterdam The Netherlands; ^4^ Patient Education Mayo Clinic Rochester MN USA; ^5^ Division of General Internal Medicine Mayo Clinic Rochester MN USA

**Keywords:** cancer communication, decision making, oncology, patient‐clinician concordance, shared decision making

## Abstract

**Background:**

Patients and clinicians do not often agree on whether a decision has been made about cancer care. This could be explained by factors related to communication quality and/or the type of decision being made.

**Methods:**

We used a self‐developed coding scheme to code a random sample of 128 encounters in which patients and clinicians either agreed (n=64) or disagreed (n=64) that a cancer care decision was made and tested for associations between concordance and key communication behaviours. We also identified and characterized cancer care decisions by topic and level of patient involvement and looked for trends.

**Results:**

We identified 378 cancer care decisions across 128 encounters. Explicit decisions were most commonly made about topics wherein decision control could be easily delegated to a clear and present expert (eg either the patient or the clinician). Related to this, level of patient involvement varied significantly by decision topic. Explicit decisions were rarely made in an observable way about social, non‐clinical or self‐management related topics, although patients and clinicians both reported having made a cancer care decision in encounters where no decisions were observed. We found no association between communication behaviours and concordance in our sample.

**Conclusions:**

What counts as a “decision” in cancer care may be constructed within disparate social roles that leave many agendas unaddressed and decisions unmade. Changing the content of conversations to encourage explicit decisions about self‐management and life context‐related topics may have greater value in enabling shared understanding than promoting communication behaviours among already high‐performing communicators.

## 1 INTRODUCTION

### 1.1 Background

Optimizing patient‐clinician communication is a priority intended to improve the quality of care in oncology and positively impact patient quality of life, patient satisfaction and medical outcomes.^1^, ^2^, ^3^, ^4^, ^5^ Shared decision‐making approaches that increase patient involvement in the decision‐making process are particularly promoted because these approaches seek to ensure that the outcomes of care‐related decisions reflect the values and preferences of patients. In previous research we showed that, when surveyed immediately after an outpatient oncology encounter, patients and clinicians often disagreed in their ratings of whether any “specific cancer care decision was made” within that encounter.^6^ This study raised questions about the quality of decision making in oncology and highlighted the possibility that patients and clinicians may define “cancer care decisions” differently.^7^ It also suggested a potential link between decision making and a broader indicator of poor quality communication long recognized in oncology, namely discordance between patients’ and clinicians’ understanding (this has been seen in regards to diagnosis, prognosis and treatment preferences, among other things).^8^, ^9^, ^10^, ^11^, ^12^, ^13^ To date, the underlying and causal communication failures that contribute to discordance in oncology are poorly understood, and our previous work shed no additional light.^6^ Identifying and describing these factors could be of value in avoiding discordance and improving communication, decision making and care quality in oncology.

Some evidence suggests that explicit communication techniques may be needed to ensure patients and clinicians remain “on the same page” and that patient‐centred goals for life and health are pursued.^14^, ^15^, ^16^, ^17^ Studies have shown, for example, that physicians often do not take the time to fully establish their patient's agenda or goals for an encounter.^18^, ^19^ In multiple medical contexts, this has been shown to lead to poor understanding of the patient's problem and desires for care by the physician.^20^, ^21^, ^22^ It is possible that, if patients and clinicians have different expectations of or agendas for an oncology encounter, these might influence their perceptions of what was or was not accomplished in it, and thus lead to discordance. Patients and clinicians could also have different perceptions of whether a “cancer care decision was made” based on their level of involvement in the decision‐making process itself. In that sense, discordance might not only be expected when patients and clinicians have different ideas about what cancer care decisions are,^7^ but also when they make independent and undisclosed decisions about their own intentions for “cancer care.” These complex issues have not been explored.

### 1.2 Objective

The objective of this study was to identify possible factors that could predict patient‐clinician concordance in perception of whether any cancer care decisions are made during oncology encounters. More specifically, we sought to assess the effects of clinicians’ efforts to, (i) elicit the patient's encounter agenda, (ii) summarize the activities of the encounter and, (iii) assess the patient's understanding of the plan. We also categorized all cancer care decisions identified by both topic and level of patient involvement and looked for trends and associations.

## 2 METHODS

### 2.1 Sample and participants

Patients and clinicians in the encounters were enrolled in a parent study.^23^ Eligible patients were at any stage of management for any solid tumour malignancy. They were being seen in their regular outpatient encounter at a large, tertiary cancer centre in the upper Midwest of the United States. Clinicians were staff oncologists, senior fellows or nurse practitioners.

### 2.2 Procedure

This study is a follow‐up analysis of our 2015 study on patient‐clinician discordance.^6^ In that study, we used survey data to identify situations in which patients and clinicians agreed (eg were concordant) or disagreed (eg were discordant) in their assessments of whether a cancer care decision was made in an outpatient oncology encounter. In the concordant sample (n=213), patients and clinicians either both responded “yes” or both responded “no” when asked if a decision was made. In the discordant sample (n=102), one responded “yes” when the other responded “no” to the same question (note that this was a “best‐case scenario” evaluation of concordance as we could not guarantee that patients and clinicians were identifying the same decisions). Surveys were collected immediately after the encounter. The encounters were audio recorded as part of the parent study, but we did not access audio recordings at the time of the 2015 study.

For this analysis, our statistician used a random sequence generator to select a random sample of 64 encounters each from both the concordant and discordant samples of the 2015 study (128 encounters in all). We estimated, using the effect size of a similar study in primary care,^22^ that this sample would give us 80% power to detect a meaningful difference in the primary outcome of whether eliciting the patient's agenda is associated with concordance at 95% confidence with a two‐sided alpha.

Recordings of the encounters were transcribed verbatim and uploaded to a web‐based data system for coding (REDCap^24^). Our research team iteratively reviewed pilot transcripts that were not part of the study sample and consulted experts to inductively develop the coding criteria for all outcomes. After calibrating judgements on the pilot sample, a team of two analysts (AL, physician researcher and cancer patient; JH, qualitative analyst experienced in patient‐clinician discourse) coded all study transcripts, while blinded to their concordance status. They coded the first 20% of the sample (26 encounters) in duplicate. After establishing interrater agreement at >75% for all codes, AL coded the remaining 80% of the encounters alone.

### 2.3 Measures

For the primary outcome, coders took note of whether the clinician made an effort to “elicit the patient's agenda.” Possible ratings were “clearly” (eg “What would you like to get out of our time today?”) “partially” (eg “What concerns or questions do you have?”) and “not at all” (eg “How are things going?”). Ratings were of clinician behaviour primarily but were outcome focused. In that sense, it was possible for encounters to be coded as “clearly” or “partially” if patients were judged to “volunteer” their agendas to that degree.

To explore other communication behaviours that might account for concordance, coders similarly took note of (i) the extent to which the clinician confirmed the course of action with the patient by restating it at the end: possible ratings were “clearly” (eg “Let me summarize what we decided on today…”) “partially” (eg “So we'll switch to drug X and see you back in…”) and “not at all” (eg “Okay, any questions for me?”); and (ii) the extent to which the clinician assessed the patient's understanding of what occurred: possible ratings were “clearly” (eg “Can you tell me what we decided to do today?”) “partially” (eg “Is everything clear? Do you have questions?”) and “not at all” (eg “Let's proceed with this plan then.”).

Simultaneously, coders flagged every instance in which a cancer care decision was made according to a set of pre‐developed criteria, and categorized each decision by topic and degree of patient involvement in making the decision (see Table**** 1 for details and categories). We developed all coding criteria after a thorough literature search and consultation with experts. We confirmed that no existing measures were feasible or appropriate for this study, often because they targeted different constructs or used conceptualizations of medical decisions that were too narrow.

**Table 1:** Decision codes and criteria

Cancer care decision criteria	
1. Arrival at a decision point where at least two alternative progressions exist	
2. One of these alternatives is selected	
3. The choice made will directly impact the medical management, health, or well‐being of the patient	
4. Was NOT a decision to postpone decision at this time	
5. Was NOT a decision to simply weigh options or a verbalization of internal negotiations	
6. Was NOT verbalization of historical decision made	
7. Was NOT information‐sharing, advice‐giving, or history‐taking	
8. A decision to pursue clear action that will lead to a decision will count (ie determine insurance and then call back with decision, etc.)	
**Potential decision topics and examples**	
Medical management	Treatment, screening, follow‐up approach
Lifestyle or behavioural changes	Diet, exercise
Personal or social plans	Change job, move in with family member
Logistical coordination	Select follow‐up location
Pursue nothing more	Hospice, end of life
CAM‐related treatments	Acupuncture, massage
Actionable step	Look at insurance, discuss with family
Seek information	Visit patient education, personal research
**Levels of patient involvement and criteria**	
No participation	No apparent participation
Agreement	Verbal agreement only
Contributor	Provides information only
Major contributor	Provides information, discusses options and plan

### 2.4 Statistical analysis

We entered all coded data from the encounters into JMP statistical software and linked it to the concordance status as recorded in the 2015 study. Only rarely did we observe “clear” and explicit examples of the communication behaviours we were assessing. As such, we elected to dichotomize all of the communication behavioural outcomes by combining ratings of “clearly” and “partially.” We then used chi‐square tests of association to evaluate the effects of clinician behaviours, setting concordance as the dependent variable. We summarized distributions with descriptive statistics and explored the data with bivariate tests of association.

## 3 RESULTS

### 3.1 Sample characteristics

Concordant and discordant samples were well balanced with regard to participant characteristics (Table 2). Overall, patients were mostly white and had a median age of 62 years (range 22‐84). The majority were in survivorship/remission or undergoing treatment for recurrent or prolonged disease. The most common tumour types were breast and gastrointestinal. Approximately 63% of the encounters were with staff oncologists (n=22), and the rest were with either senior fellows (n=8) or nurse practitioners (n=5) (note that, consistent with the 2015 study, no clinicians were disproportionately more prevalent in either the concordant or discordant encounters).

**Table 2:** Sample characteristics

Encounter characteristic	Concordant encounters (n=64)	Discordant encounters (n=64)	Total encounters (n=128)
Patient gender female (%)	39 (61)	32 (50)	71 (55)
Patient median age (range)	62 (34‐83)	62 (22‐84)	62 (22‐84)
Patient race (%)			
Asian	0 (0)	0 (0)	0 (0)
Black/African Descent	0 (0)	1 (2)	1 (1)
Indian/Native Native	0 (0)	1 (2)	1 (1)
White/Caucasian	64 (100)	61 (95)	125 (98)
Other	0 (0)	2 (3)	2 (2)
Patient tumour type (%)			
Brain	4 (6)	2 (3)	6 (5)
Breast	19 (30)	15 (23)	34 (27)
Gastrointestinal	15 (23)	22 (34)	37 (29)
Genitourinary	4 (6)	6 (9)	10 (8)
Gynecological	6 (9)	3 (5)	9 (7)
Head/Neck	3 (5)	2 (3)	5 (4)
Lung	8 (13)	5 (8)	13 (10)
Melanoma	2 (3)	2 (3)	4 (3)
Sarcoma	3 (5)	6 (9)	9 (7)
Unknown	0 (0)	1 (2)	1 (1)
Patient cancer care stage (%)			
Initial diagnosis	1 (2)	3 (5)	4 (3)
Early initial treatment	5 (8)	3 (5)	8 (6)
Mid initial treatment	14 (22)	11 (17)	25 (20)
Post‐treatment/survivorship	20 (31)	28 (44)	48 (38)
Recurrence, on treatment	22 (34)	16 (25)	48 (30)
End Stage	3 (5)	4 (6)	7 (5)
Clinician type (%)			
Staff oncologist (n=22)	40 (62)	41 (64)	81 (63)
Oncology fellow (n=8)	14 (22)	10 (16)	24 (19)
Nurse Practitioner (n=5)	10 (16)	13 (20)	23 (18)
Clinician median age (range)	39 (29‐67)	43 (29‐67)	40 (29‐67)

### 3.2 Communication behaviours

Only rarely did we observe “clear” and explicit examples of the patient‐centred communication behaviours we were assessing (n=7 instances for agenda elicitation, n=26 for encounter summarization and n=2 for assessing understanding). We found no significant association between any of the behaviours and patient‐clinician concordance in perception. Rather, the behaviours were actually observed with greater frequency in the discordant encounters. For example, agendas were elicited to some degree in 16 of the concordant encounters and 22 of the discordant encounters (*P*=.33), while assessment of patient understanding occurred in 18 of the concordant encounters and 28 of the discordant encounters (*P*=.10). Restatement and summarization of the encounter's activities occurred with high frequency in both the concordant and discordant sample, being noted in 50 and 54 cases, respectively (Table 3).

**Table 3:** Frequency of communication behaviours in oncology encounters

Observed patient‐centred communication behaviour	Concordant encounters (n=64)	Discordant encounters (n=64)	Fisher's *P*
Elicitation of patient agenda?			
Clearly or partially	16	22	.33
Not at all	48	42	
Restatement or summary of encounter?			
Clearly or partially	50	54	.50
Not at all	14	10	
Assessment of patient understanding?			
Clearly or partially	18	28	.10
Not at all	46	36	

### 3.3 Cancer care decisions

We identified and coded 378 unique cancer care decisions across the 128 encounters. Encounters comprised, on average, three decisions each, although seven encounters had none and one encounter had nine. The vast majority of the decisions were related to medical management (73%) and included issues related to treatment, follow‐up and testing, while 17% of the decisions were related to logistical coordination issues (eg the geographic location at which the patient would receive treatment) (Table 4). Although discussion related to some of the other topics did occasionally occur, explicit decisions were rarely made.

**Table 4:** Types of decisions made and level of patient involvement

Characteristics of observed decisions	Decisions in concordant encounters (n=200) (%)	Decisions in discordant encounters (n=178) (%)	Total observed decisions (n=378) (%)
Decision topic			
Medical management	141 (70)	135 (76)	276 (73)
Seek info and education	1 (1)	1 (1)	2 (1)
Pursue nothing more	0 (0)	2 (1)	2 (1)
Personal or social	9 (5)	1 (1)	10 (3)
Logistical coordination	33 (17)	30 (17)	63 (17)
Lifestyle/behavioural	7 (4)	3 (2)	10 (3)
Actionable step	9 (5)	6 (3)	15 (4)
CAM	0 (0)	0 (0)	0 (0)
Patient involvement level			
None	14 (7)	19 (11)	33 (9)
Agreement	84 (42)	71 (40)	155 (41)
Contributor	56 (28)	47 (26)	103 (27)
Major contributor	46 (23)	41 (23)	87 (23)

Patients were major contributors to decisions in 23% of cases. Most commonly, clinicians would state a course of action and the patient would make a verbal utterance of agreement, such as “okay” or “yes” (41% of cases). Patients were noted to have no participation in 9% of all cancer care decisions made. The level of patient involvement was significantly associated with the type of decision being made (chi‐square *P*<.0001). Patients were major contributors in 59% of all logistical decisions, for example, but had no participation in or agreed only with 60% of all medical management decisions (Table 5).

There were no significant differences in decision characteristics by encounter concordance status. Two hundred decisions occurred within concordant encounters, while the rest (n=178) were in discordant. Concordance remained low even at the extremes of number of decisions made within encounters. For example, in the seven encounters where no decisions were made, patients and clinicians agreed that no decision was made in only three cases. In three other cases, the clinician reported that a decision was made when the patient did not. In the 7th case, the opposite was true. Similarly, patients and clinicians did not frequently agree that any decisions were made in encounters where even six or more decisions were observed (n=8).

**Table 5:** Variation in patient participation across decision topic

Topics w 10 or more instances	No participation (%)	Agreement (%)	Contributor (%)	Major contributor (%)
Actionable step (15)	2 (13)	7 (47)	4 (27)	2 (13)
Lifestyle/behavioural (10)	1 (10)	1 (10)	5 (50)	3 (30)
Logistical (63)	0 (0)	11 (17)	15 (24)	37 (59)
Personal social (10)	0 (0)	1 (10)	6 (60)	3 (30)
Medical management (276)	30 (11)	134 (49)	73 (26)	39 (14)

## 4 DISCUSSION AND CONCLUSION

### 4.1 Discussion

We sought to explore the effects of patient‐centred communication behaviour and decision‐making factors on the development of shared understanding in oncology. We were unable to detect an association between any of the communication behaviours we evaluated and patient‐clinician concordance in the assessment of whether a decision was made. These null results should be considered in the context of the body of evidence in support of these behaviours, however, and in the light of key limitations of the present study. Indeed, the underlying measure of concordance in our study relied on a “best‐case scenario” analysis that could not know whether patients and clinicians agreed that the same decision had been made in an encounter. The magnitude of this effect was not considered in the power analysis and, thus, the study may be significantly underpowered. It is also possible that the patients and clinicians in our sample might have been uniformly high‐performing communicators (the patients were all highly educated and had high health literacy). If they were, the explicit behaviours we evaluated could have been either too simplistic or of little incremental value in facilitating a detectable difference in the development of shared understanding. Of course, our findings could also be explained by differences in patients’ and clinicians’ ideas about what “cancer care” is and by our inability to observe and measure the internal deliberations, thoughts and experiences of the participants. In some cases, for example, patients and clinicians reported that decisions were made in encounters in which we identified none (despite using a very inclusive coding criteria). Because the underlying survey item asking patients to rate whether a decision had been made had been pre‐tested and made cognitive sense to both patients and clinicians, we can be reasonably certain that patients and clinicians were accurately reporting their thoughts about when a cancer care decision had been made in an encounter.

In total, we identified and categorized 378 decisions in oncology by topic and level of patient involvement. Our findings are consistent with others^25^ in highlighting the complexity of oncology encounters. Overall, we found that patients were highly involved in decisions that required logistical coordination of care around their individual lives and circumstances. Involvement appeared to be limited, however, in decisions related to medical management. Although medical management is certainly a topic area in which clinicians have special expertise, opportunities to increase patient involvement likely exist. Indeed, models of person‐centred care^26^, ^27^ aim to individualize treatment plans to the unique contexts, needs and preferences of patients. Implicitly, this requires clinicians to obtain contextual information about their patients that could inform treatment modification. This level of involvement would have been categorized as “contributor” or “major contributor” in our analysis and was not seen in the majority of medical management decisions. We also found very few explicit decisions related to potentially relevant but “extra‐clinical” topics (eg social, personal, lifestyle, CAM) that are consistent with a person‐centred approach to care.

Overall, confidence in the validity of our results is elevated because we used a prospective analysis with blinded coders. Key limitations relate to the homogeneity of the study sample, potentially low variation in the communication behaviours of clinicians, uncertainty about the construct validity of the survey item used to assess concordance, and the fact that the majority of transcripts were coded by one individual. Aside from this, this study represents, to our knowledge, one of the largest and most rigorous analyses of patient‐centred communication and decision making in oncology.

### 4.2 Conclusion

In summary, we showed that explicit decisions were often only made in relation to topics where there appeared to be a clear and present expert (either the patient or the clinician). We also showed that patients and clinicians may make undisclosed and isolated decisions about the very care they are supposed to be optimizing together in partnership. In order for communication to act as a promoter of person‐centred care, targeted clinician and/or patient education may be required. Specifically, the patient‐clinician interaction may need to be reshaped to encourage more open and intentional dialogue. Indeed, as this research highlights, efforts to optimize the communication behaviours of clinicians may reach a plateau in their effectiveness. If this is true, achieving the next stage of value from patient‐centred communication may require a change in the content of what gets deliberated (as opposed to the quality only). For this to occur, it appears patients and clinicians both may need to be more open with one another and to reserve space for shared decisions related to topics about which no clear and present expert (clinician or patient) exists.

### 4.3 Practice implications

The importance of communication in oncology has long been recognized.^28^ Although high quality patient‐clinician communication is accepted as a central component of patient‐centred care, the exact ways in which it is able to contribute to improved health outcomes are typically unclear.^29^ In this exploratory study, we showed that there may be more opportunity to improve *what* gets talked about rather than *how* it is discussed. Specifically, we found opportunities to expand oncology discussions through the pursuit of more explicit, shared decisions about topics in which uncertainty is great and in which patient context, preference and belief system are especially relevant. To the extent these topics affect patient desires, efforts and abilities for self‐care, it is not difficult to imagine pathways through which their inclusion could affect health outcomes.

Still, further study is needed to evaluate the meaning and significance of our findings, ideally in other settings, with larger samples, and through different methods. Inductive, qualitative study could be especially helpful, particularly in attempting to understand what patients and clinicians are counting as “decisions.” Practically, it is important to understand the feasibility and opportunity costs of changing the discussion in oncology practice. Indeed, efforts to fully align agendas, elicit contextual information and increase patient and clinician involvement require time. Integrating these activities into the care delivery system (without sacrificing discussion that oncology clinicians must prioritize to ensure that care is meeting safety and efficacy targets) will be challenging. This may justify the development and trying of conversation aids that encourage discussion and decisions related to more diverse “cancer care”‐related topics.

## CONFLICT OF INTEREST

All authors declare that they have no conflicts of interest.

## AUTHOR'S CONTRIBUTIONS

AL conceptualized this study and conducted all the coding in partnership with JH and with oversight from JT. AL oversaw the statistical analysis and wrote the initial draft of the manuscript, with substantive support from CF, VM and JT. MK provided substantial editorial feedback and assisted in developing the final draft. All authors approved the final draft.
